# Resolution of PMA-Induced Skin Inflammation Involves Interaction of IFN-*γ* and ALOX15

**DOI:** 10.1155/2013/930124

**Published:** 2013-06-02

**Authors:** Guojun Zhang, Xiaoman Liu, Chunhui Wang, Liwei Qu, Jingjing Deng, Hui Wang, Zhihai Qin

**Affiliations:** ^1^Research Center for Immunology, Xinxiang Medical University, 601 Jinsui Road, Xinxiang, Henan 453003, China; ^2^Institute of Biophysics, Chinese Academy of Sciences, 15 Datun Road, Beijing 100101, China; ^3^Graduate School of the Chinese Academy of Sciences, No.19A Yuquan Road, Beijing 100049, China

## Abstract

*Background.* Acute inflammation and its timely resolution play important roles in the body's responses to the environmental stimulation. Although IFN-*γ* is well known for the induction of inflammation, its role in the inflammation resolution is still poorly understood. *Methodology and Principal Findings.* In this study, we investigated the function of interferon gamma (IFN-*γ*) during the resolution of PMA-induced skin inflammation *in vivo*. The results revealed that the expression levels of IL-6, TNF-*α*, and monocyte chemoattractant protein 1 (MCP-1) in skin decreased during the resolution stage of PMA-induced inflammation, while IFN-*γ* is still maintained at a relatively high level. Neutralization of endogenous IFN-*γ* led to accelerated reduction of epidermal thickness and decreased epithelial cell proliferation. Similarly, decreased infiltration of inflammatory cells (Gr1^+^ or CD11b^+^ cells) and a significant reduction of proinflammatory cytokines were also observed upon the blockade of IFN-*γ*. Furthermore, neutralization of IFN-*γ* boosted ALOX15 expression of the skin during inflammation resolution. In accordance, application of lipoxin A4 (LXA4, a product of ALOX15) obtained a proresolution effect similar to neutralization of IFN-*γ*. These results demonstrated that through upregulating ALOX15-LXA4 pathway, blockage of IFN-*γ* can promote the resolution of PMA-induced skin inflammation.

## 1. Introduction

Resolution of acute inflammation plays an important role during host defense to pathogenic microorganisms as well as during the body's response to trauma, tissue injury, ischemia/reperfusion, and surgical interventions [1, 2]. Inflammation resolution is considered as a proactive process that terminates inflammatory responses, promotes tissue recovery, and returns to homeostasis [3]. Delayed resolution of acute inflammation can cause chronic inflammation, which underlies the pathology of several chronic inflammatory disease processes, such as inflammatory bowel disease, rheumatoid arthritis, asthma, and psoriasis [4].

Kinds of immune cells and cytokines contribute to the resolution of inflammation. Normally, the resolution phase of inflammation is characterized by the orderly phagocytic clearance of apoptotic granulocytes and debris by macrophages [5]. T regulatory cells (Tregs), formerly known as suppressor T cells, can also contribute to the resolution of inflammation [6]. Among inflammation-resolving cytokines, IL-10 and TGF-*β* are the most important mediators. However, the mechanism that controls resolution of acute inflammation has not been fully described and is still under investigation.

IFN-*γ* is a key mediator of inflammatory responses [7]. It has antivirus effects [8], directly activates macrophages, enhances antigen presentation [9], and promotes the differentiation of cytotoxic T cells [10]. IFN-*γ* also plays an important role in many inflammatory diseases, such as obesity [11], experimental autoimmune encephalomyelitis [12], and chemical-mediated inflammation such as PMA-induced skin inflammation and papilloma [13]. Previous study showed that IFN-*γ* was directly involved in and strongly enhanced papilloma development during the promotion stage by upregulating several key proinflammatory cytokines and the T helper 17 (Th17) response [13]. On the other hand, UVB-induced neonatal skin melanocyte activation can be abolished by antibody-mediated systemic blockade of IFN-*γ* [14]. Although there is now emerging evidence indicating that IFN-*γ* acts as a master regulator of immune responses and inflammation, the function and the mechanism of IFN-*γ* in the resolution of acute inflammation are not fully understood.

Diverse classes of oxygenated lipids contribute to the active resolution of inflammation. Lipoxins (LXs) are a kind of eicosanoid mediator that possesses a wide spectrum of potent anti-inflammation and proresolving effects [15]. It is reported that LXs can suppress neutrophil chemotaxis, inhibit vascular dilatation, and decrease vascular permeability. In addition, LXs can promote emigration of monocytes and their ingestion of apoptotic neutrophils [16]. Other lipid mediators such as Resolvin E1 and protectin D1 can also facilitate phagocyte removal of dead and dying cells by regulating leukocyte infiltration, increasing macrophage ingestion of apoptotic polymorphonuclear neutrophils *in vivo* and *in vitro* [17]. Currently, it is not clear whether IFN-*γ* influences the synthetic process of lipid mediators in the resolution of inflammation.

PMA is the most commonly used phorbol ester to induce epidermal thickening and dermal inflammation. By employing PMA-induced acute inflammation model in this study, we found that IFN-*γ* can prevent the resolution of PMA-induced skin inflammation by the downregulation of ALOX15-LXA4 pathway. This provides new insight into the role of IFN-*γ* during resolution of acute inflammation.

## 2. Materials and Methods

### 2.1. Animals

IFN-*γ*R deficient and wild-type 129/Sv/Ev mice were inbred strains purchased from The Jackson Laboratory. All mice were maintained in a specific pathogen-free environment. Sex- and age-matched mice were used with the approval of appropriate authorities.

### 2.2. Skin Inflammation Induction and Resolution

PMA (4 *μ*g, Sigma, USA) was painted on the dorsal skin of mice twice a week for 3 times and then stopped for the resolution of PMA-induced skin inflammation. Mice were sacrificed 3 hours, 4 days, or 7 days after the third PMA treatment for histological evaluation of the skin inflammation resolution.

### 2.3. Neutralization of IFN-*γ* *In Vivo*

To block the IFN-*γ* activity *in vivo*, 3 hours, 24 hours, and 4 days after the third PMA treatment, mice were injected intraperitoneally with 0.5 mg rat anti-mouse IFN-*γ* monoclonal antibody (mAb, R4-6A2) or an isotype-matched control mAb (rat IgG) as control.

### 2.4. Application of LXA4 and BOC2

LXA4 (Cayman, 100 ng per mouse) and BOC2 (GenScript, 10 *μ*g per mouse) were dissolved in PBS buffer and intraperitoneally injected into the mice 3 hours after the third PMA treatment and then every two days.

### 2.5. Hematoxylin and Eosin (HE), Sirius-Red/Fast-Green, and Immunofluorescence Analysis

Preparation of cryostat or paraffin tissue sections and immunostaining were done as described previously [18]. After HE staining, the thickness of the epidermis (in micrometers) was measured using an image system (Photoshop) and calculated as follows: actual thickness of epidermis = on-screen measurements of epidermis/magnification (5 fields per section and sections from more than 3 mice were observed). For collagen fiber detection, the sections were stained with Sirius-Red and Fast-Green (Sigma) and rinsed with 0.5% acetic acid in PBS. For immunofluorescence analysis, sections were stained with rat anti-mouse CD11b (1 : 100; BD Pharmingen, USA), rat anti-mouse granulocyte differentiation antigen 1 (Gr1, 1 : 100; BD Pharmingen, USA), rabbit anti-mouse fibroblast-specific protein-1 (FSP1, 1 : 100; BD Pharmingen, USA), and subsequently rhodamine-labeled secondary antibody and counterstained with 4, 6-diamidino-2-phenylindole (DAPI; Sigma, USA). As for Brdu staining, mice were intraperitoneally injected with 2 mg Brdu (Sigma) 5 hours before sacrifice. Skin sections were stained with mouse anti-Brdu (Zhongshan Goldenbridge, Beijing) to display the proliferating cells. Immunofluorescence of tissue sections was evaluated on an fluorescence microscopy (Olympus).

### 2.6. Preparation of Skin Homogenates and Determination of Cytokine Concentrations

Mice were sacrificed and the dorsal skin was removed, weighed, and homogenized in ice-cold Tris-EDTA buffer. The supernatant was determined using a mouse inflammation cytometric bead array (CBA) kit (BD Pharmingen, USA) for the levels of IL-6, MCP-1, IFN-*γ*, and TNF-*α* and using a cytokine mouse Luminex System for IL-1*α* and IL1-*β*. The level of LXA4 was detected by a ELISA kit (Neogen).

### 2.7. Real-Time PCR

Total RNA was extracted from mouse skin using Trizol (Tiangen Biotech, Beijing). cDNAs were synthesized using the Reverse Transcription System (Promega, USA). Real-time reverse transcription PCR was done using a Corbett real-time PCR detection system (Corbett Rotor-Gene). Specific mRNA level of each sample was normalized to the respective GAPDH mRNA. The levels of gene expression in the skin of mice without PMA treatment were rated as 1 arbitrary unit and used as a baseline to compare expression levels of the same gene in different samples. Designed primers were as follows: GAPDH, 5′-CATCAAGAAGGTGGTGAAGC-3′ and 5′-CCTGTTGCTGTAGCCGTATT-3′; Alox15, 5′-GCGACGCTGCCCAATCCTAATC-3′, and 5′-CATATGGCCACGCTGTTTTCTACC-3′; Alox12, 5′-AGACAACAGACCTACTGCTGG-3′, and 5′-TCCTTACAGTCCGCTGCTATT-3′; and COX2, 5′-CTCCCTGAAGCCGTACACAT-3′ and 5′-ATGGTGCTCCAAGCTCTACC-3′.

### 2.8. Statistical Analysis

Data were analyzed using two-tailed unpaired Student's *t*-test or one-way ANOVA. Means ± SD were presented. Statistically significant differences are indicated as follows: *P < 0.05.

## 3. Results

### 3.1. The Expression of IFN-*γ* Is Maintained at a High Level, While the Expression of Other Cytokines Decreased during the Resolution Stage of PMA-Induced Skin Inflammation

**Figure 1:**
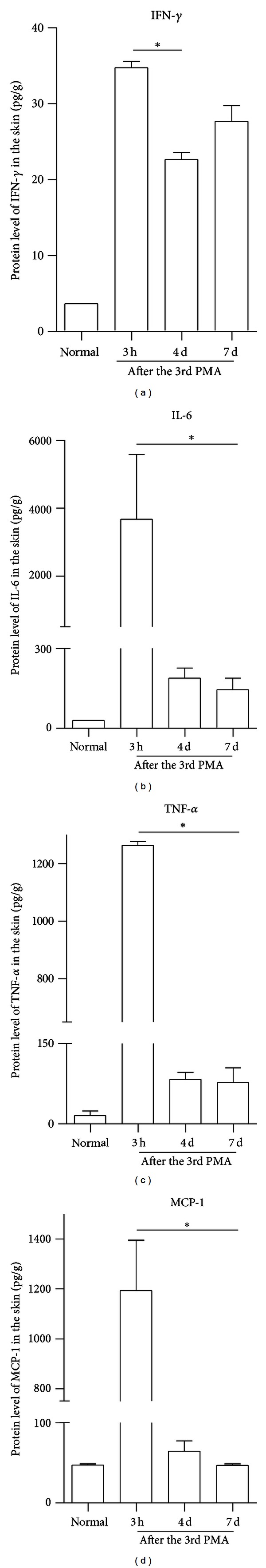
IFN-*γ* expression in the skin maintained at a relatively high level during the resolution stage of PMA-induced inflammation. PMA was painted to the dorsal skin of mice (*n* = 3 per group) for 3 times (twice a week) and then stopped for the resolution of inflammation. Mice were sacrificed 3 hours, 4 days, or 7 days after the third PMA treatment. The skins were removed, weighted, and homogenized in ice-cold Tris-EDTA buffer. The concentrations of IFN-*γ* (a), IL-6 (b), TNF-*α* (c), or MCP-1 (d) in tissue supernatants were then detected at the indicated times. *P < 0.05, compared to the level of 3 hours after the 3rd PMA treatment.

PMA is often used as a promoting agent in “two-stage” carcinogenesis protocol [19]. PMA administration causes the rapid onset of cutaneous edema accompanied by significant leukocyte infiltration and inflammatory cytokine secretion. We and many other researchers had studied the role of IFN-*γ* in inflammation and tumor progression, but few focused on the function of IFN-*γ* in the resolution of acute cutaneous inflammation. Therefore, we employed the PMA-induced acute skin inflammation model. After 3 times PMA treatment (twice a week), an obvious dry and swelling skin could be observed, and then by stopping PMA treatment the skin became thinner and smoother in one week. The concentrations of IFN-*γ* and other pro-inflammatory cytokines including IL-6, TNF-*α*, and MCP-1 were detected during the skin inflammation resolution stage (Figure 1). The results showed that the expression levels of all the four cytokines were significantly enhanced 3 hours after the third PMA treatment, which confirmed their pro-inflammatory effect. Four or seven days after the third PMA treatment, the expression levels of IL-6, TNF-*α*, and MCP-1 were all greatly decreased compared with those of hour 3. In contract, IFN-*γ* concentration slightly decreased 4 or 7 days after the third PMA treatment but still stayed at a relatively high level compared to that of the other cytokines. The result indicated a special role of IFN-*γ* in the resolution of acute skin inflammation.

### 3.2. Blockade of IFN-*γ* Promoted the Resolution of PMA-Induced Skin Inflammation

**Figure 2:**
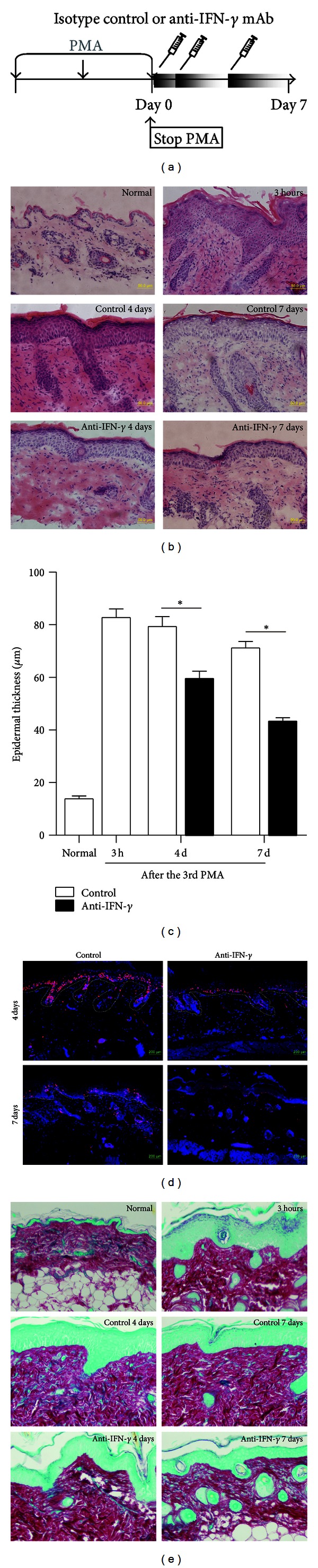
Neutralization of endogenous IFN-*γ* led to accelerated reduction of epidermal thickness and epithelial cell proliferation. (a) Mice were treated with isotype control mAb (control) or anti-IFN-*γ* mAb (anti-IFN-*γ*) after the third PMA treatment. Then, mice from control and anti-IFN-*γ* groups were sacrificed 3 hours, 4 days, or 7 days after the third PMA treatment. (b) HE-stained skin sections of different groups were shown. Statistical analysis of the epidermal thickness of skin from normal (without PMA treatment), control, or anti-IFN-*γ* groups was measured as described in Methods (5 fields per section, 5 or 6 mice per group) and presented in (c) Scale Bar, 50 *μ*m. *P < 0.05, compared to control group. (d) Skin sections were stained with anti-Brdu (red) to display the proliferating cells. The boundary between epidermis and dermis is marked by a dotted line. Scale Bar, 200 *μ*m. (e) Skin sections of different groups were stained with Sirius-Red binding to all types of collagen and Fast-Green binding to noncollagenous proteins (magnification as (b)).

To investigate the role of IFN-*γ* in the resolution stage of skin inflammation, we neutralized endogenous IFN-*γ* after the third PMA treatment for one week (Figure 2(a)). The epidermal thickness and proliferation of epithelial cells were measured in this week. As shown by HE staining in Figure 2(b), numerous leukocytes infiltrated to the PMA-treated skin even at the resolution stage. However, when endogenous IFN-*γ* was neutralized, the number of infiltrating leucocytes deceased as compared with that of control group. The epidermal thickness of IFN-*γ*-neutralized mice was significantly reduced than that of control mice no matter 4 days or 7 days after the third PMA treatment (*P* < 0.05) (Figure 2(c)). The BrdU incorporation study showed that there were less proliferating cells in the skin of IFN-*γ*-neutralized mice than those in the control mice with 4 or 7 days' recovery (Figure 2(d)). Meanwhile, the collagen fibers in the skin were analyzed by Sirius-Red/Fast-Green staining to show the process of inflammation resolution, but no significant difference was observed between control and anti-IFN-*γ* groups (Figure 2(e)). The results indicated that blockade of IFN-*γ* facilitated resolution of PMA-induced skin inflammation.

### 3.3. Neutralization of Endogenous IFN-*γ* Reduced Inflammatory Cell Infiltration and Inflammatory Cytokine Production in the Skin

**Figure 3:**
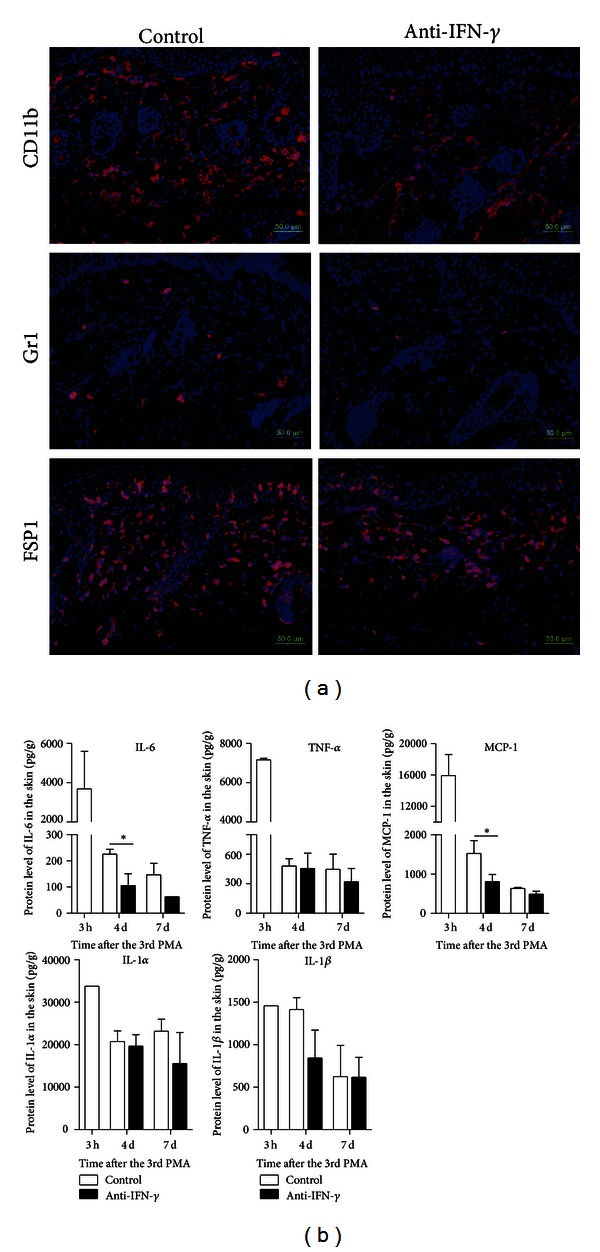
Neutralization of endogenous IFN-*γ* decreased the infiltration of inflammatory cells and the production of inflammatory cytokines in the skin. Mice were sacrificed 3 hours, 4 days, or 7 days after the third PMA treatment. (a) Skin sections were stained with anti-CD11b, anti-Gr1, or anti-FSP1 (red). Nucleoli were stained with DAPI (blue). Scale Bar, 50 *μ*m. (b) The expression levels of IL-6, TNF-*α*, MCP-1, IL-1*α*, and IL-1*β* of the skin from control group or anti-IFN-*γ* group were detected at indicated time points. *P < 0.05, compared to control group.

To further clarify the pro-resolving effect of IFN-*γ* neutralization, we investigated the cell subsets in the resolving skin. Four days after the third PMA treatment, there were less Gr1^+^ cells or CD11b^+^ cells in the skin of IFN-*γ*-neutralized mice compared with that in control group, but no significant difference was observed in the numbers of FSP1^+^ cells of different groups (Figure 3(a)). The proinflammatory cytokines in the inflamed skin were also detected. Compared with that of control group, IL-6 concentration in IFN-*γ*-neutralized group was decreased by 52% on day 4 after the third PMA treatment and by 53% on day 7. Decreased levels of MCP-1 and IL-1*β* were also observed in IFN-*γ*-neutralized mice on day 4 and IL-1*α* on day 7 after the third PMA treatment. However, neutralization of IFN-*γ* did not affect the expression level of TNF-*α* in the resolving skin (Figure 3(b)). These results demonstrated that neutralization of endogenous IFN-*γ* decreased the infiltration of Gr1^+^ cells or CD11b^+^ cells and reduced the production of IL-6, MCP-1, and IL-1*β*.

### 3.4. Neutralization of IFN-*γ* Boosted the mRNA Expression of ALOX15 and LXA4 Generation in Skin at the Resolution Stage of PMA-Induced Inflammation

**Figure 4:**
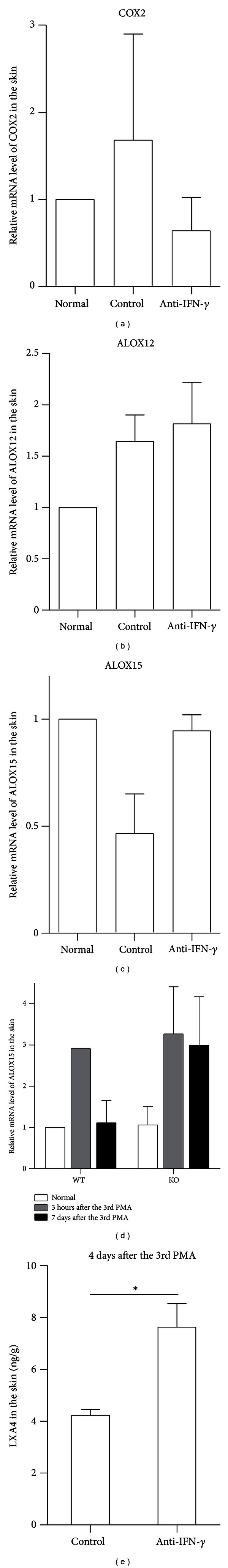
Neutralization of endogenous IFN-*γ* promoted the ALOX15 expression of the skin. Mice from control or anti-IFN-*γ* group were sacrificed 4 days after the third PMA painting. The skin was removed, and the mRNA level of COX2 (a), ALOX12 (b), and ALOX15 (c) was detected by real-time PCR. (d) IFN-*γ*R knockout (KO) and wild-type 129/Sv/Ev (WT) mice were sacrificed 3 hours or 7 days after the third PMA treatment. The mRNA level of ALOX15 in the skin was measured by real-time PCR. (e) The levels of LXA4 in skin of control and anti-IFN-*γ* groups 4 days after 3 times PMA treatment were detected by ELISA. *P < 0.05.

The metabolism of arachidonic acid (AA) plays important role not only in the onset, but also in the resolution process of inflammation. COX2, ALOX12, and ALOX15 are important enzymes for the metabolism of AA and for the promotion or resolution of inflammation [20]. Thus we next focused on these important enzymes to explore the effect of IFN-*γ* on skin inflammation resolution. The mRNA levels of these enzymes in skin were detected 4 days after the third PMA treatment. The results revealed that the expression of COX2 mRNA of the skin was elevated after PMA treatment, which was coincident with the former research by Park et al. [21]. But COX2 expression attenuated with the neutralization of IFN-*γ* (Figure 4(a)). The expression of ALOX12 mRNA in PMA-treated mice was similar to that in mice with neutralization of IFN-*γ* (Figure 4(b)). The expression of ALOX15 mRNA was inhibited by the PMA treatment, and neutralization of IFN-*γ* rescued the ALOX15 expression to a similar level as that of the normal group (Figure 4(c)).

To confirm the effect of endogenous IFN-*γ* on the expression of ALOX15 during the resolution of acute skin inflammation, we treated wild-type mice and IFN-*γ* receptor knockout mice (IFN-*γ*R-KO) with PMA as described previously. It is found that ALOX15 mRNA expression was elevated in IFN-*γ*R knockout mice at the resolution stage compared with that of wild-type mice (Figure 4(d)). ALOX15 plays a critical role in the synthesis of LXA4 which is a metabolite from AA and the first lipid mediator found to have a proresolution role [15]. Moreover, a higher level of LXA4 was detected in the skin of anti-IFN-*γ* group than that of control 4 days after 3 times PMA painting (Figure 4(e)). These results indicated that ALOX15 and LXA4 were involved in the process of anti-IFN-*γ*-mediated inflammation resolution.

### 3.5. Application of LXA4 Has a Pro-Resolution Effect as Neutralization of IFN-*γ*

**Figure 5:**
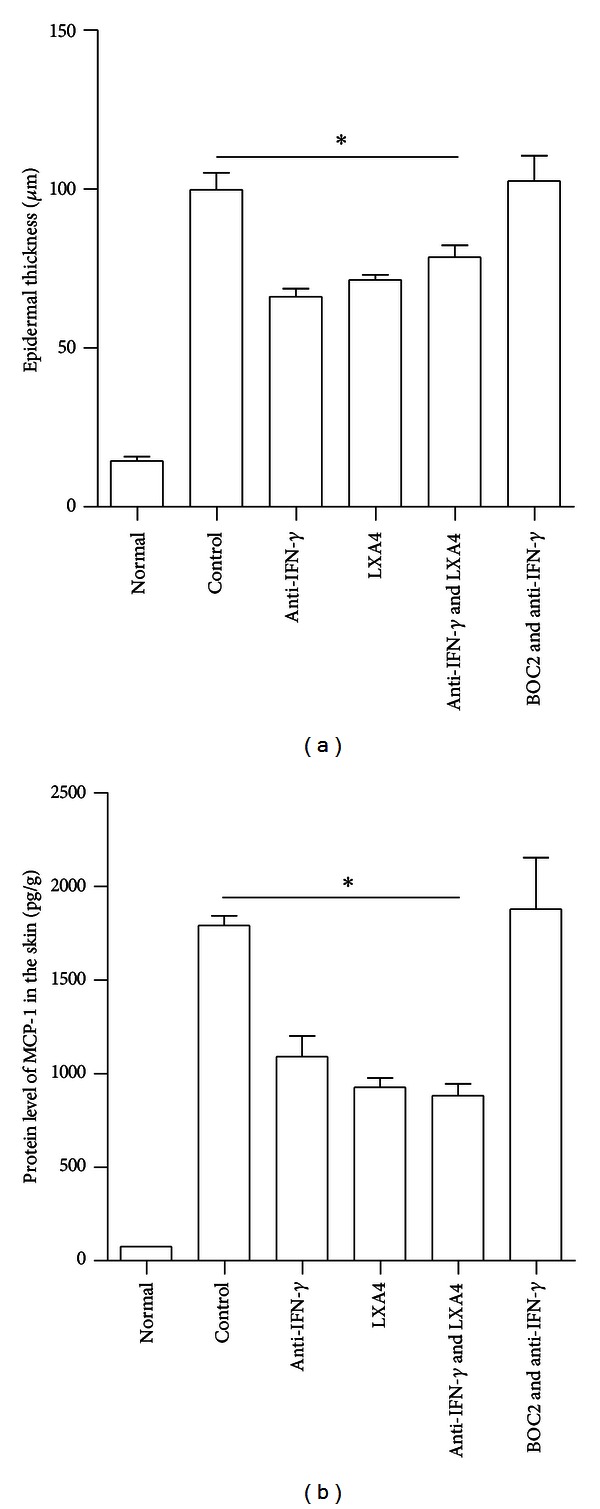
Application of LXA4 obtained a pro-resolution effect, while blocking LXA4 reverted the effect of IFN-*γ* neutralization. Three hours after the third PMA painting, mice were divided into 5 groups: control, anti-IFN-*γ*, LXA4, anti-IFN-*γ* and LXA4, and BOC2 and anti-IFN-*γ*. The skin tissues were removed 4 days after the third PMA treatment. Epidermal thickness (a) was analyzed by HE staining as described in Methods. The MCP-1 expression level (b) in the skin was detected by CBA. *P < 0.05, compared to control group.

To confirm the involvement of ALOX15-LXA4 pathway in the effecting process of anti-IFN-*γ* treatment at skin inflammation resolving stage, we treated mice with LXA4 and analyzed the thickness of the skin in the resolution stage. It is shown that the skin thickness of LXA4 group was significantly reduced compared to the control group and so was the group with both LXA4 and IFN-*γ* neutralization antibody treatment. When BOC2, a LXA4 receptor antagonist [22], was given to IFN-*γ*-neutralized mice, the epidermal thickness was reverted to the same level as that in control group (Figure 5(a)). In addition, the concentration of MCP-1 was decreased in mice treated with LXA4 or anti-IFN-*γ* antibody separately or combined, in comparison with that in control mice. BOC2 application in IFN-*γ*-neutralized mice elevated the MCP-1 expression to the same level as that in control mice (Figure 5(b)). All these results indicate that blockage of IFN-*γ* promoted skin inflammation resolution through an ALOX15-LXA4-associated pathway.

## 4. Discussion

Inflammation can be fired by traumatic, infectious, or toxic injury anywhere in the body. Topical PMA administration can induce acute skin inflammation. Many cytokines participate in the progress including IFN-*γ*. IFN-*γ* was thought to promote the development of inflammation during the early stage. Our previous result revealed that IFN-*γ* expression was elevated greatly 3 hours after PMA administration, and this confirmed the pro-inflammation role of IFN-*γ*. In addition, IL-6, TNF-*α*, and MCP-1 participated in the early pro-inflammatory stage of PMA-induced acute inflammation. During the resolving stage (4 days and 7 days after the third PMA treatment), the protein level of IL-6, TNF-*α*, and MCP-1 decreased remarkably, but IFN-*γ* still sustained at a high level. This suggested that IFN-*γ* may have different role from IL-6, TNF-*α*, and MCP-1 during the resolving stage of inflammation. This speculation encouraged us to explore the function of IFN-*γ* during the resolution of inflammation.

PMA-induced skin inflammation was often used for detecting anti-inflammatory effects of all kinds of drugs [23–25]. Skin inflammation can be evoked by topical PMA application, with increased vascular permeability, edema, swelling, and then substantial inflammatory cell infiltration within the dermis and epidermal hyperplasia [26]. Increasing skin thickness is the hallmark of skin irritation and local inflammation. Otherwise, leukocyte infiltration and release of inflammatory cytokines were considered as the other two marks of inflammation. PMA treatment twice a week for 3 times can induce acute skin inflammation [27].

Usually acute inflammation resolve in relatively short time. However, if assisted repair is not properly phased, acute inflammation can develop to protracted inflammation that leads to persistent tissue damage by leukocytes, lymphocytes, or collagen [2]. Inflammatory resolution is an active, highly regulated process already encoded at the onset of inflammation and required to prevent the transition into chronic inflammation associated with spreading of tissue injury and exacerbated scarring [28]. Multiple mechanisms normally ensure resolution. Cells like macrophages switch phenotypes from pro- to anti-inflammatory, and additional mediators of resolution arise, including proteins, lipids, and gasses [14]. Epidermal thickness was the ultimate certification of inflammatory resolution. Our data showed that IFN-*γ*-neutralized mice had thinner epidermis, less proliferating epithelial cells, reducing Gr1^+^ cells and CD11b^+^ cells and lower level of IL-6 and MCP-1 than control mice during skin recovery (4 days and 7 days after the third PMA treatment). Consistently, Ishida et al. showed that IFN-*γ* deficiency can accelerate the wound healing process. Compared with WT mice, IFN-*γ* KO mice or mice with anti-IFN-*γ* antibody exhibited an enhanced angiogenesis, accelerated collagen deposition, and then rapid wound closure and granulation tissue formation after punch biopsy in the back skin [29]. All these results supported that neutralization of endogenous IFN-*γ* can promote the recovery of inflamed skin.

The lipoxins were the first identified and recognized endogenous anti-inflammatory lipid mediators which can function as “braking signals” for neutrophils or chalones in inflammation [30]. LXA4 was one of the native lipoxins, which were products of the lipoxygenase pathway produced from AA [31]. Arachidonate 15-Lipoxygenases (ALOX15) and Arachidonate 12-Lipoxygenases (ALOX12), members of the LOX family that oxygenate free polyenoic fatty acids, were two key enzymes to synthesize LXA4 [32]. Research for murine model of acute peritonitis has shown that ALOX12-ALOX15-deficient eosinophils have no effect on promoting inflammation resolution [33]. Cyclooxygenase2 (COX2) is an inducible enzyme associated with inflammation [34]. COX2 expression was elevated in mouse epidermis treated with PMA [35].

Was the rapid resolution of skin inflammation by neutralization of IFN-*γ* linked to the LXA4? We estimated the expression of COX2, ALOX12, and ALOX15, important enzymes regulating LXA4 synthesis. The results showed that neutralization of IFN-*γ* inhibited the elevation of COX2 expression induced by PMA but enhanced the expression of ALOX12 and ALOX15, which may result in increasing LXA4 synthesis. Upregulation of ALOX15 mRNA in IFN-*γ*R knockout mice during resolution confirmed the reinforcement of LXA4 synthesis by IFN-*γ* deficiency. LXA4 blocking experiments *in vivo* showed that suppression of LXA4 function by BOC2 restored not only the skin thickness, but also the concentration of MCP-1 of the skin to the level of control. The above results further confirmed that the effect of IFN-*γ* deficiency in inflammation resolution was exerted by the way of ALOX15-LXA4. There are still some questions that need further investigations. According to others' work and the infiltrated cell populations in this model, ALOX15 may be expressed in macrophages [36, 37] and fibroblasts [38]. In another work, we showed that the mRNA expression of ALOX15 can be detected in macrophages from tumor bearing mice and skin fibroblasts from neonatal mice, and IFN-*γ* can downregulate ALOX15 expression in these cells *in vitro* [39]. However, it is still unknown in which cells *in vivo* that ALOX15 is expressed and regulated by IFN-*γ*. As for the expression of LXA4 receptor, it is reported that it can be expressed by not only leukocytes, but also endothelial cells, fibroblasts, and epithelial cells [40–42]. More work has to be added to investigate the targets of LXA4 in the skin inflammation model.

In this study, we have shown that IFN-*γ* deficiency can expedite resolution of PMA-induced skin inflammation. Considering the enhancement of IFN-*γ* neutralization to ALOX15, we speculated that IFN-*γ* inhibits the resolution of PMA-induced skin acute inflammation by the possible way of downregulation of ALOX15-LXA4 pathway.

## 5. Conclusions and Significance

The data showed that neutralization of IFN-*γ* facilitated the resolution of PMA-induced skin inflammation by up-regulation of ALOX15-LXA4 pathway. The regulation of IFN-*γ* and LXA4 may have a potential therapeutic effect for treating acute skin inflammation.
